# Universal linguistic hierarchies are not innately wired. Evidence from multiple adjectives

**DOI:** 10.7717/peerj.7438

**Published:** 2019-08-01

**Authors:** Evelina Leivada, Marit Westergaard

**Affiliations:** 1 Department of Language and Culture, UiT The Arctic University of Norway, Tromsø, Norway; 2 Department of Language & Literature, NTNU Norwegian University of Science and Technology, Trondheim, Norway

**Keywords:** Language, Cognition, Innateness, Development

## Abstract

**Background:**

Linguists and psychologists have explained the remarkable similarities in the orderings of linguistic elements across languages by suggesting that our inborn ability for language makes available certain innately wired primitives. Different types of adjectives, adverbs, and other elements in the functional spine are considered to occupy fixed positions via innate hierarchies that determine orderings such as A>B>C, banning other permutations (*B>C>A). The goal of this research is to tap into the nature and rigidity of such hierarchies by comparing what happens when people process orderings that either comply with them or violate them.

**Method:**

*N* = 170 neurotypical, adult speakers completed a timed forced choice task that featured stimuli showing a combination of two adjectives and a Spelke-object (e.g., ‘I bought a square black table’). Two types of responses were collected: (i) acceptability judgments on a 3-point Likert scale that featured the options ‘correct’, ‘neither correct nor wrong’, and ‘wrong’ and (ii) reaction times. The task featured three conditions: 1. size adjective > nationality adjective, 2. color adjective > shape adjective, 3. subjective comment adjective > material adjective. Each condition had two orders. In the congruent order, the adjective pair was ordered in agreement with what is traditionally accepted as dictated by the universal hierarchy. In the incongruent order, the ordering was reversed, thus the hierarchy was violated.

**Results:**

In the first experiment, the results of *n* = 140 monolinguals showed that across conditions, both congruent and incongruent orders were generally accepted as correct. For 2/3 conditions, the difference in acceptability ratings between congruent and incongruent orders did not reach statistical significance. Using time as a window to processing, reaction times showed that incongruent orders do not take longer to process than congruent ones, as should be the case if the former were treated as being licensed under some type of special condition (e.g., contrastive focus) that reverses the unmarked order and legitimizes the violation of the hierarchy. In the second experiment, the results of *n* = 30 bidialectals, tested in both language varieties, corroborated the findings of the first experiment.

**Conclusions:**

Our findings do not provide evidence for an innate hierarchy for adjective ordering that imposes one rigid, unmarked order. We discuss the importance of notions such as subjectivity and inherentness, and show that for some conditions, not only is there no evidence for a hard constraint that bans incongruent orders, but even simple preferences of congruent orders over incongruent ones are hard to discern. Capturing the bigger picture, given that both the hierarchies and their legit permutations have been described as innate, our results reduce the amount of primitives that are cast as innate, eventually offering a deflationist approach to human linguistic cognition.

## Introduction

Linguists have often noted that linguistic elements are arranged in a strikingly uniform way across typologically different languages (Rizzi, 1997; Rizzi, 2004; Cinque, 1999; Belletti, 2004; Laenzlinger, 2005; Shlonsky, 2010; Rizzi & Cinque, 2016). This line of investigation gradually gave rise to what came to be known as the *cartographic approach*. As a research endeavor with the aim to draw maps of syntactic configurations that are as detailed as possible (Cinque & Rizzi, 2008), cartography has been an influential protagonist in the field of theoretical linguistics over the last decades. Research in psychology and clinical linguistics has also generated dominant hypotheses that rest on the assumption that the building blocks of syntactic computation occur in specific and highly detailed arrays of functional particles (e.g., the Tree-Pruning Hypothesis, according to which agrammatic aphasics produce certain blocks of the functional hierarchy that are intact up to a node but pruned past it; Friedmann & Grodzinsky, 1997; Martínez-Ferreiro, 2010). In an effort to grant experimental support to cartography, it has been argued that neurolinguistic and psycholinguistic experiments “provide a rich source of evidence with which to corroborate linguistic theory” (Garraffa, 2007: 10). At the same time, such experiments offer a way of tapping into human linguistic cognition, because linguists have explained the cross-linguistic similarities that characterize the ordering of certain elements through supporting the idea of a *common source*: the attested orders fall out from hierarchies that are part of our innate endowment for language and as such species-universal. To explain this further with a concrete example, it has been observed that adjectives —among other types of linguistic elements—show a remarkably similar pattern of placement across various languages: (1a)–(3a) are standardly assumed as acceptable, while (1b)–(3b) are not.

**Table:** 

(1a)	a big black bag.
(1b)	#/*a black big bag.^1^
(2a)	un coche rojo grande. [Spanish]
	*a car red big*
	‘A big red car.’
(2b)	#/*un coche grande rojo.
	*a car big red*
	(intended meaning) ‘A big red car.’
(3a)	ena megalo mple vivlio. [Greek]
	*a big blue book*
	‘A big blue book.’
(3b)	#/* ena mple megalo vivlio.
	*a blue big book*
	(intended meaning) ‘A big blue book.’

The purportedly 1Throughout the introduction, we use the symbol ‘#’ to mark expressions which in the literature have been presented as semantically anomalous and ‘*’ to mark expressions which have been argued to be unacceptable and grammatically ill-formed. Our own judgments may differ.universal order for adjective placement has been argued to boil down to a syntactic hierarchy of closeness to the noun (4) that is encoded in Universal Grammar (Cinque, 1994; Scott, 2002; Panayidou, 2013; Cinque & Rizzi, 2008; Rizzi & Cinque, 2016), which stands for the innate endowment for language within generative linguistics (Chomsky, 1981).

**Table:** 

(4)	Subjective Comment > Evidential > Size > Length
	> Height > Speed > Depth > Width > Temperature > Wetness > Age
	> Shape > Color > Nationality/Origin > Material (adapted from Scott, 2002: 114)

In addition to proposals that endorse a syntactic origin for ordering restrictions, the literature includes a number of proposals that support a cognitive origin. These proposals attribute cross-linguistic similarities in adjective ordering to cognitive notions such as inherentness (Whorf, 1945), absoluteness (Sproat & Shih, 1991) and subjectivity (Hetzron, 1978; Scontras, Degen & Goodman, 2017) in the following way: more objective/absolute adjectives that denote noun-inherent properties are placed closer to the noun. Inherentness refers to how intrinsic to the noun the property denoted by the adjective is, absoluteness measures the degree to which the adjective attribute is absolute or relative, and subjectivity refers to how likely faultless disagreement over the attribute is. For example, the color adjective in (5) denotes a property that is less subjective/more absolute than the one denoted by the size adjective which in turn expresses a property that is usually understood as less subjective/more absolute compared to the property expressed by evaluative adjectives such as *beautiful* (but see Scontras, Degen & Goodman, 2017 for a more nuanced approach to subjectivity and to how size adjectives may not reliably differ from evaluative adjectives).

**Table:** 

(5)	A beautiful big white mural.

Different communicative purposes can lift the ordering restrictions. For instance, contrastiveness requires an interpretation that is based on a marked form such as focus fronting ((6); Panayidou, 2013). Restrictions are also lifted in augmented noun phrases, as is the case with Determiner Spreading in Greek (i.e., a grammatical phenomenon whereby each adjective is accompanied by a determiner (7); Alexiadou, 2001; Alexiadou & Wilder, 1998). Adjectives separated by ‘comma intonation’ also allow freer order ((8); Martin, 1969; Sproat & Shih, 1991; Teodorescu, 2006).

**Table:** 

(6)	I asked you to bring me the BLUE big book, not the green one.
(7a)	to megalo to mple to vivlio. [Greek]
	*the big the blue the book*
	‘The big blue book.’
(7b)	to mple to megalo to vivlio.
	*the blue the big the book*
	‘The big blue book.’
(8)	The big, wonderful, pink roses.

Importantly, contexts that lift ordering restrictions do *not* host the basic unmarked order, when they deviate from (4). Put another way, regardless of the origin of the ordering constraints one endorses (i.e., grammatical or cognitive), there is consensus that only *one* order can be the unmarked one and that a phrase such as ‘a red big car’ should not be considered well-formed in the absence of any special context (e.g., focus), comma intonation, or some other type of licensing of the deviation from the hierarchy. The cognitive accounts can allow for some ordering flexibility for a given multi-adjective string, *if* the two adjectives are equally inherent or subjective. At present, the predominant theory (or, at least, one of the most prominent ones) is that an innately encoded hierarchy such as the one in (4) dictates adjective orderings in a universal fashion. At the same time, an increasing number of studies argue that resorting to an innate endowment to account for certain orders is merely stipulative and carries little explanatory power ((Svenonius, 2008; Bouchard, 2012; Leivada, 2015; Bacskai-Atkari, 2018); see also Ramchand & Svenonius, 2014 on an attempt to derive the tense-verb hierarchy rather than cast it as a Universal Grammar primitive). Last, it is worth noting that even works that spot the limited strength of the cartographic approach on the ‘explanatory power’ front, still embrace a good deal of its theoretical premises or predictions. For example, Svenonius (2008) argues that Universal Grammar dictates structures that constrain ordering in a way that makes size adjectives consistently precede color, origin/nationality, and material adjectives.^2^ 2The combination of a size adjective and a nationality/origin adjective is one of the combinations that our experiment puts to test.This embracing of some of the basic premises of cartography that occurs even after realizing its limited explanatory strength is perhaps the most powerful indication of how influential the cartographic approach —that assigns a rich structure to Universal Grammar and takes hierarchies to be innately wired—has been and continues to be in linguistics.

Recent corpus studies have cast further doubt on the predictions of (4). For example, Kotowski & Härtl (2019) document naturalistic data that show size adjectives preceding subjective comment/evaluative adjectives or color adjectives preceding shape adjectives. Both these orderings violate (4), given that they were not featured in a context that would justify lifting ordering restrictions. At the same time, Kotowski & Härtl’s (2019) overall results do show the presence of one hard constraint: in their corpus, relational adjectives (i.e., non-gradable adjectives that typically derive from nouns and express a relation between the noun on which they are formed and the one they modify; for example, material in ‘wooden toy’) are always found closer to the noun than other adjectives. Incidentally, origin and material are the two categories that occupy the bottom positions of the hierarchy of closeness to the noun in (4), hence this finding is compatible with the hierarchy put forth in the cartographic approach too. These results raise the question: Are we dealing with hard constraints, simple preferences, a mix of both, or neither?

To sum up, there are two topics that are under debate with respect to adjective orderings, and our research taps mainly into question (I) and indirectly into question (II):

(QI) Is there a universal hierarchy for adjective ordering that —in the absence of any special licensing that lifts restrictions—gives rise to an unmarked order?

(QII) Do ordering restrictions, to the degree they are attested, correspond to hard constraints or to mere preferences?

A number of recent approaches to adjective ordering aim to derive possible restrictions (Truswell, 2009; Kotowski, 2016; Scontras, Degen & Goodman, 2017; Simonič, 2018; Hahn et al., 2018; Kotowski & Härtl, 2019), without taking them for granted under the assumption that they are dictated by (4). Truswell’s (2009) corpus results suggest that the cartographic approach undergenerates with respect to the adjective orderings that are attested in actual language data: intersective adjective pairs allow free order (e.g., ‘square purple box’ and ‘purple square box’), while the ordering in pairs that feature an intersective and a subsective adjective of the nominal is less free (e.g., ‘big wooden table’ but *‘wooden big table’).^3^ 3Intersective adjectives make a contribution that results in an intersection of what the nominal and the adjective denote. For example, if one is an *American* surgeon and is also an activist, then it is a valid conclusion that one is an American activist. When subsective adjectives combine with a nominal, the composition returns a subset of the denotation of the nominal in the following way: if one is a *skillful* surgeon and a dancer, it does not follow that one is a skillful dancer (see Kennedy, 2012 for an introduction to the semantic properties of each class).

Using a different way to approach adjective ordering constraints, Kennison (2010) reports the results of two self-paced reading tasks, where participants read the stimuli in a word-by-word fashion by pressing a key. The first task tapped into the ordering between a color adjective and a size adjective, while the second task featured a color adjective and an adjective that denoted subjective comment (e.g., ‘beautiful’). In both tasks, the tested stimuli involved two conditions. In the Adjective Order Violation Condition, participants read sentences like ‘John /said that /the red /big /balloon /was /the most /expensive /one. /’, which violates (4). In the Preferred Adjective Order Condition, the adjective ordering complied with (4) and size preceded color: ‘John /said that /the big /red /balloon /was /the most /expensive /one. /’. With respect to the three critical regions (i.e., adjective 1, adjective 2, and noun), Kennison (2010) reports that longer times were observed in the Violation Condition compared to the Preferred Condition. A review of her methodology and results suggests two important things: first, at the time participants received the second adjective (which was the most critical region because it established the alleged violation), they did not yet know whether the sentence would end with a strategy that would legitimize this violation (see (6)) or not. Phrased differently, there was no violation per se at that point. The longer reading times in the Violation Condition may be the outcome of a processing cost that arises when one mentally constructs contexts that justify deviations from what is the most frequently attested/expected pattern. Second and more important, Kennison (2010) argues that the difference between the two conditions was statistically significant in the most critical region (i.e., region 4 that featured the second adjective) of the second task at *p* = 0.03. Given that many regions were compared, if one corrects for multiple comparisons, it is not clear whether the significance survives. Even if one does not correct for multiple comparisons, the classical *p* = 0.05 threshold does not offer black or white proof of evidence: *p* = 0.04 and *p* = 0.06 represent a quite similar state of affairs when translated on a continuous measure (Hamel & Gilles Yoccoz, 2018). Recent initiatives to revise the standards for statistical significance along the lines of a continuum have suggested that the values between approximately 0.10 and 0.05 offer inconclusive evidence, the values between approximately 0.05 and 0.01 offer weak to moderate evidence, while the values below approximately 0.01 offer convincing evidence (Ramsey & Schafer, 2013). Other scholars place the corresponding thresholds for moderate and convincing evidence at 0.005 and 0.001 respectively, as a remedy for solving the irreproducibility problem of scientific research (Johnson, 2013). For these two reasons, we interpret Kennison’s (2010) results as showing, at best, weak evidence for ordering restrictions.

Returning to the literature on adjective ordering, we want to mention Kotowski (2016), who employs a multimethod approach to adjective ordering restrictions, combining a corpus study with psycholinguistic testing. His results suggest that ordering restrictions are not grammatical phenomena, represented syntactically, but the outcome of a complex interplay between factors such as morphophonological weight, the idiomaticity of certain combinations of nominals and adjectives (cf. the example of Svenonius, 2008: *wild rice* is a species of rice; this combination of an adjective and a nominal forms an idiom that does not relate in any way to the typical properties of wildness), or the notion of polysemy/ambiguity that may dress an adjective with different meanings. Scontras, Degen & Goodman (2017) carried out a number of experiments to adduce strong evidence that explains the observed ordering preferences under an account that is based on general properties of cognition, and more specifically, on the notion of subjectivity. Kotowski & Härtl (2019) offer a pragmatic account that capitalizes on the role of norms and generalizations in linguistic input.

Evidently, the literature on adjectives has recently started questioning the viability of the cartographic approach. At the same time, the latter remains influential in current accounts of adjective ordering. A number of recent works follow standard cartographic assumptions in accepting the adjective hierarchy to be innate and universal (Sheehan, 2017; Huang, 2017), in acknowledging it as one of the possible explanations on the topic of adjective ordering (Payne, 2018; Trotzke & Wittenberg, 2019), or in developing proposals that put forth less fine-grained hierarchies that are, however, quite similar to those explored in cartography (Pereltsvaig & Kagan, 2018). In sum, it has been recently argued that it is still unclear which theory is the most adequate to explain the attested data (Trotzke & Wittenberg, 2019), while several attempts to experimentally verify the basic claims of cartography in relation to adjective ordering are currently in progress (Mišmaš, Marušič & Žaucer, 2018; Plesničar, 2018; Dolenc, 2018). Fitting the purposes of the third group of studies, the present work aims to add to the several recent works that have produced results that call the cartographic claims into question (e.g., Scontras, Degen & Goodman, 2017; Kotowski & Härtl, 2019).

Rather than focusing on the origin of ordering restrictions, the present work takes a crucial step back and examines their very existence. The research aim behind the present experiment is thus to address (QI), by tapping into the rigidity of the predictions made by syntactic approaches to adjective ordering restrictions, specifically approaches that take such restrictions to fall out from innately wired hierarchy such as the one in (4).

## Materials & Methods

The research aim is approached in two ways through an off-line and an on-line measure. The off-line measure is an acceptability judgment rating using a 3-point Likert scale, which examines whether there are differences in the acceptability of orders that either violate or adhere to the hierarchy (henceforth, incongruent orders and congruent orders respectively) in (4). The on-line measure records reaction times and determines whether the processing of incongruent orders is costlier, possibly due to an effort to compute and license a marked order. Reaction times are sensitive to the number of operations and alternative meanings that must be computed (Bates et al., 1982). In empirical terms this translates into reaction times that are shorter in the processing of canonical/unmarked orders, due to the fact that deviations from the unmarked order induce an extra processing cost (Erdocia et al., 2009). The unmarked order has a lower processing cost and this happens because the cognitive parser forms expectations about forthcoming constituents based on its experience about what is the most frequently encountered option (Imamura, Sato & Koizumi, 2016 and references therein). In other words, there is an association between frequency and processing cost: the more frequent an order is, the easier it is for the parser to engage with it and process it in a shorter time. Deviations from the expected/preferred order clash with what the parser anticipates, hence the increase in the reaction time. In the domain of adjective ordering, this predicts an additional processing cost in deriving the interpretation of the incongruent orders (see also Plesničar, 2018).

We used Ibex Farm (Drummond, 2013) in order to run two timed forced choice experiments that used the same task, administered in Standard Greek for the monolingual group and in both Standard and Cypriot Greek for the bidialectal group. This task measured acceptability judgment ratings in monolingual, neurotypical, adult speakers of Standard Greek (*n* = 140; mean age = 37.08, SD = 11.06; see Table 1 for demographic information) and bidialectal, neurotypical, adult speakers of Standard and Cypriot Greek (*n* = 30; mean age = 32, SD = 11.28; see Table 2 for demographic information). The results of the two groups are presented separately as Experiment 1 and Experiment 2 respectively.

**Table 1:** Demographic information for monolinguals (experiment 1).

**Participants**		
Gender	Male	66
	Female	74
Education	Secondary	18
	Tertiary	122
Handedness	Right	126
	Left	14

**Table 2:** Demographic information for bidialectals (experiment 2).

**Participants**		
Gender	Male	11
	Female	19
Education	Secondary	6
	Tertiary	24
Handedness	Right	28
	Left	2

All participants provided informed consent prior to their involvement in the study, in accordance with the Declaration of Helsinki. The Norwegian Center of Research Data reviewed and approved the study protocol (approval reference number: 55775 / 3 / LH). Excluding criteria for participants included non-native knowledge of Greek or native knowledge of other languages (assessed on the basis of self-report), reception of speech-pathology treatment and presence of neurological/neurodegenerative diseases (based on self-report), and inappropriate behavior in ungrammatical fillers (i.e., accepting them). Single automatic responses below 600 ms (i.e., when a button is pressed too fast, without allowing enough time for actual consideration of the presented stimuli) were also excluded, while more than two automatic responses led to the exclusion of the participant.

In experiment 1, the task involved three conditions of the following adjective pairs: 1. size-nationality, 2. shape-color, 3. subjective comment-material. Each condition had two orders with three test structures per order (18 test structures in total). In the congruent order, the size adjective preceded the nationality adjective, in agreement with the hierarchy in (4). In the incongruent order, the hierarchy was violated and the adjective ordering was reversed. Since reaction times were measured, all test structures were carefully matched for length and syntactic structure across orders and conditions. All adjectives appeared in direct object position and featured a combination of two adjectives and a Spelke-object, that is, a concrete object that maintains its connectedness and boundaries when moving. Examples of the actual test items in Standard Greek are given in (9)–(10) for the congruent and the incongruent order. Standard Greek has been argued to restrict ordering in a way that conforms to the hierarchy in (4) (Stavrou, 1999; Alexiadou, 2003). The ordering has been argued to be strict (Alexiadou, 2003) and based on notions such as absoluteness, eventually boiling down to an opposition between subject/speaker-oriented and object-oriented adjectives, which differ in terms of their subjectivity (Stavrou, 1999).

**Table:** 

(9)	Iða ena tetragono mavro trapezi. (congruent)
	*see.PAST one square black table*
	I saw a square black table
(10)	Pira ena malino omorfo fustani. (incongruent)
	*get.PAST one woollen beautiful dress*
	I got a woolen beautiful dress

Experiment 2 consisted of two tasks. The first task was the one presented above for experiment 1. The second task was an adaptation of the first task in Cypriot Greek. We used different test items, but the same methodology and design. This combination of tasks in experiment 2 enabled us to have 36 observations per participant, 18 for each variety. The order of presentation of the two tasks in terms of which variety was tested first was pseudorandomized across participants. Cypriot Greek, being a non-standard variety that lacks the status of an official language, is still underinvestigated in various domains of grammar. Adjective ordering is one of them; to the best of our knowledge, there is no theoretical or experimental work that discusses the existence of adjective ordering restrictions in Cypriot Greek. On the basis of the obtained results which did not show any difference in the performance in the two varieties, the results of both tasks are presented together in the section Results of Experiment 2 and are contrasted to those obtained from the group tested in the first experiment.

Following the experimental design of Stowe & Kaan (2006), in both tasks the ratio of fillers to actual test structures was 2:1, while the ratio of grammatical stimuli to ungrammatical stimuli, the latter referring to ungrammatical fillers, was 1:1. The stimuli did not involve ambiguous adjectives, meaning that an adjective could not have two different interpretations in the phrase in which it occurred. Morphophonological length/weight was not controlled for, but was taken into account in the design. In experiment 1, all adjective pairs were 6 syllables long (3+3, 4+2 or 2+4). In the pairs of the condition size-nationality, the average syllables were 3.16+2.83 for adjective 1 and adjective 2 respectively. In the condition shape-color, the average was 2.83+3.16, while in the condition subjective comment-material, the average was 3+3. If two adjectives are interchangeable in terms of syntactic order, morphophonological length/weight may play a role in placement, with a preference for the lengthier adjective to be placed closer to the noun (Kotowski, 2016). One could thus argue that length/weight may influence acceptability judgment patterns in the incongruent orders: if the syntactic-order preference clashes with the preference to place the lengthier adjective closer to the noun, an order deviating from the hierarchy may eventually be rendered acceptable because the latter preference is stronger. For this reason, we also calculated the average syllables per pair in the incongruent orders alone. In the pairs with incongruent order in the condition size-nationality, the average syllables were 4+2, in the condition shape-color the corresponding average was 2.3+3.6, in the condition subjective comment-material it was 3.3+2.6, while the overall average when merging incongruent orders across the three conditions was 3.22+2.77, with the shorter adjective being closer to the noun. This means that, if the incongruent orders are judged as acceptable, these averages would argue against interpreting length/weight as the mitigating factor.

In experiment 1, lemma frequency was consistently >1 for content words in all test structures (checked through GreekLex2; Kyparissiadis et al., 2017). Lemma frequency was not calculated for the second task of experiment 2, because no word frequency data are available for Cypriot Greek. With respect to the unacceptable stimuli (i.e., the ungrammatical fillers), their ungrammaticality was due to violations of ordering constraints, but none of these constraints was related to adjective ordering. (11) shows an example of an ungrammatical filler.

**Table:** 

(11)	*The little boy ate the all candies (instead of “The little boy ate all the candies”).

The order of presentation was pseudorandomized across conditions. The full tasks and the two obtained datasets are openly available at dataverse.no/dataset.xhtml?persistentId=doi:10.18710/NTLLUF.

Participants received the sentences one by one and were asked to press a key, judging the well-formedness of each sentence on a 3-point Likert scale that involved the following options: (i) correct, (ii) neither correct nor wrong, (iii) wrong. It was explained to them prior to their participation in the study that we sought to determine how the sentences sounded to them, and not whether they are deemed correct according to grammar books. Participants did not have the option to skip a test item or go back and change an answer they gave in a previous question. Before the actual task started, a warm-up session familiarized participants with placing three fingers on keys ‘1’ (wrong), ‘2’ (neither correct nor wrong) and ‘3’ (correct) and selecting an answer without moving the entire hand, thus minimizing noise in reaction time. Concerns about the latter was the reason behind choosing a 3-point Likert scale instead of a higher-point one, despite the fact that higher-point scales have been associated with higher reliability (Preston & Colman, 2000): With three options, participants were easily trained to press the relevant keys, keeping three fingers placed on them (i.e., two fingers for the dominant hand).

With respect to the design of the tasks, it is worth mentioning that no context or background information was given to the participants. This aspect of the design is important in two ways. First, given that the order of presentation of test structures was pseudorandomized across participants, there was a balance between whether a congruent or an incongruent order was encountered first when completing the task. Also, given the high number of fillers and the restrictions we imposed on the sequence of randomized blocks, test structures of the same order and condition never appeared consecutively, hence minimizing potential priming or other extragrammatical effects that could have an impact on the judgments. In this sense, the present design is different from the one recently developed in Scontras, Degen & Goodman (2017), where participants were simultaneously presented with a congruent and an incongruent order and were asked to choose which of the two sounded more natural. In that study, certain ordering preferences were demonstrated to exist in participants’ performance, but the design was such that participants did not judge the two possible orders in isolation. This does not challenge the results of Scontras, Degen & Goodman (2017), because their methodology aimed at testing *preference*, not *acceptability*, which is what the present study tests. Therefore, their results are informative with respect to what order participants prefer when they have both orders available but are not directly informative as to how acceptable the incongruent order is. A simultaneous juxtaposition of the two orders does not tap into whether speakers truly accept an incongruent order such as ‘red small chair’, but into the strength of the preference that may arise over its explicit comparison with the prescriptively correct ‘small red chair’. Put differently, it is possible that when speakers are simultaneously presented with a congruent and an incongruent order, they do not provide the same type of unbiased judgment with respect to *acceptability*, as they do when they encounter the incongruent order alone. As the literature on acceptability judgment tasks has repeatedly suggested, the notion of prescriptive correctness can interfere with acceptability, as acceptable sentences may be rejected either on a prescriptive basis or because they are formulated in a way that is not the most natural/preferred one for the informant (Tremblay, 2005).

The second reason behind this out-of-context presentation of test stimuli is that we seek to determine whether any ordering restrictions truly exist in an *unmarked* grammatical context and discourse setting, that is, in the absence of any background information and/or special context that would legitimize lifting the restrictions. Put another way, forming an incongruent (i.e., marked) order that deviates from (4) creates the implicature of a specific contextual condition that legitimizes this deviation, and as Kotowski & Härtl (2019) argue, this implicature cannot be resolved in out-of-context presentation of stimuli. This means that in the design we employ, there is nothing to legitimize any deviation from the hierarchy. As a consequence, the default unmarked order should be left as the *only* acceptable option in the performance of our participants, *if* indeed such a single, unmarked order exists, and all other permutations require special licensing. The design of the present study is thus aptly fit to address research question (I), which is the backbone of this research.

## Results of Experiment 1

As is often the case with experiments that measure reaction times, some automatic responses were recorded and eliminated. With respect to acceptability judgment ratings, no data were eliminated from congruent orders and 0.07% of the data were eliminated from incongruent orders. Only automatic responses were removed. With respect to reaction times, 0.27% of the data were eliminated from congruent orders and 0.35% from incongruent orders. In the case of the on-line measure, apart from automatic responses, these percentages include long reaction times that were removed after data normalization with minimal a priori trimming (following the treatment advocated in Baayen & Milin, 2010). Reaction times did not follow a normal distribution due to a skewed right tail, therefore the standard logarithm (*RT*′ = log_10_(*RT*)) was applied to normalize the data and a classical ±3SD filter was then applied to detect outliers. The removed data did not alter the significance of the results; the same statistically significant associations are observed before and after data normalization and outlier trimming.

**Figure 1:**
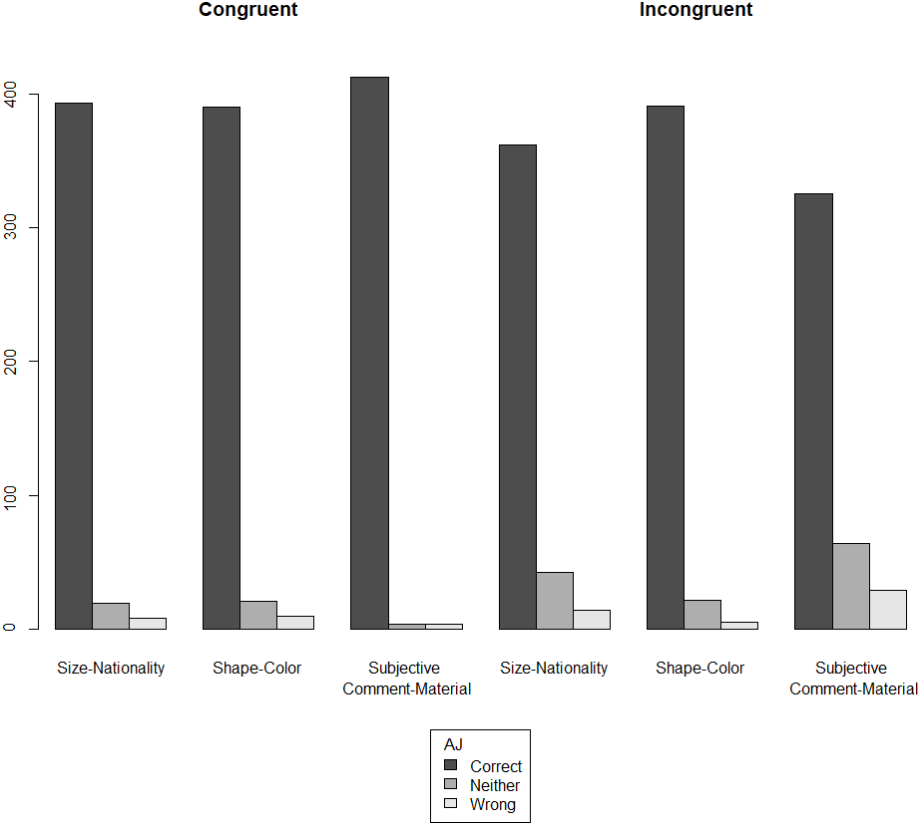
Acceptability judgment (AJ) ratings across orders and conditions in monolinguals (140 participants ×18 test items).

Starting with the off-line measure, we observe that participants largely accept the incongruent orders across the three conditions: size-nationality, shape-color, and subjective comment-material. As shown in Fig. 1, for both orders, in all three conditions, the judgment ‘correct’ is the predominant response.

**Figure 2:**
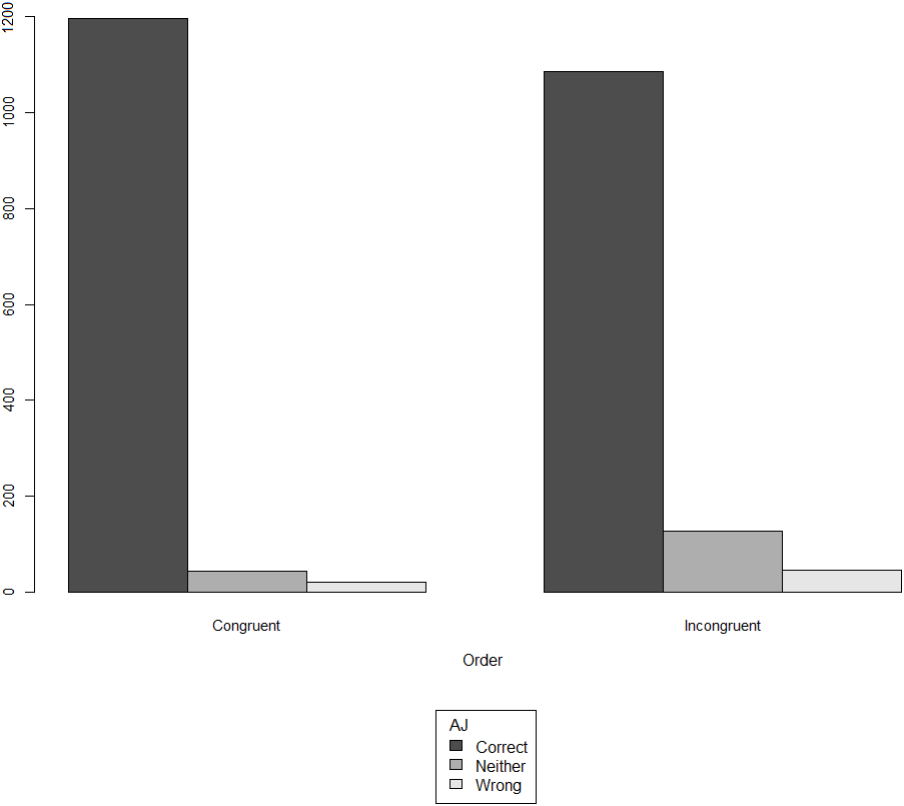
Acceptability judgment (AJ) ratings in monolinguals, split by congruency.

Figure 2 presents the overall results of the acceptability judgment ratings, split on the basis of order (congruent vs. incongruent), having merged the three conditions.

Examining Figs. 1 and 2 together, perhaps the most striking result of our experiment is the *uniformity* that is observed across the congruent and the incongruent orders in terms of the assigned ratings of acceptability. In both orders, the same pattern of acceptability is shown: the most frequent judgment is ‘correct’, followed by ‘neither correct nor wrong’, leaving ‘wrong’ as the least frequent evaluation. This pattern of acceptability is of course unsurprising in the congruent orders: for instance, subjective comment adjectives are indeed acceptable when they precede material adjectives (e.g., ‘a pretty wooden necklace’ instead of ‘a wooden pretty necklace’). However, our results further suggest—contra a long line of literature that supports the hierarchy given in (4)—that deviating from this order does *not* alter this pattern of acceptability. In other words, violating (4) does not give rise to a sentence that the majority of our participants considers wrong in the realm of their linguistic repertoire.

We analyzed the acceptability judgment ratings in two ways. The reason for this is that the literature involves mixed views on whether Likert scale data should be treated through parametric statistics or not. On the one hand, it has been argued that descriptive statistics and parametric tests give unclear results when applied to Likert scale responses (Kuzon, Urbanchek & McCabe, 1996), with the recommendation of using chi-squared tests and other non-parametric analyses for such responses. On the other hand, recent research has suggested that parametric statistics can be applied to Likert scale data (Sullivan & Artino, 2013), and in the field of linguistics specifically, experiments that use Likert scales to tap into acceptability judgments have shown the use of ANOVA to be as reliable as that of more sophisticated tests such as Random Forests and Regression and Classification Trees (Endresen & Janda, 2016). At the same time, non-parametric tests are less powerful in comparison to parametric tests and require a bigger sample size (Sullivan & Artino, 2013). Since our sample is relatively large, we analyze our data first as categorical through chi-squared tests and then as ordinal through ANOVAs. In the second analysis, we treated the acceptability judgment ratings as clusters of numbers (in line with (Endresen & Janda, 2016), adding 3 for each time a participant gave the judgment ‘correct’, 2 for ‘neither correct nor wrong’, and 1 for ‘wrong’, thus obtaining an overall score for each test item.

A generalized linear model showed the effect of condition to be significant in the dataset treating the dependent variable as multinomial (*χ*^2^ = 16.02, *p* = 0.003), hence we performed individual analyses of congruent-incongruent orders in each condition. Treating the data as categorical, three chi-squared tests were run, comparing ratings in the congruent and incongruent orders: one for each condition. At a confidence level of 99%, *α* = 0.0033 after Bonferroni correction for multiple comparisons (i.e., three comparisons). Taking the null hypothesis to be that there is no difference between congruent and incongruent orders, this hypothesis can be rejected only in condition 3, subjective comment-material, where the difference between the ratings assigned to the congruent orders and the ones of incongruent orders appears statistically significant: *χ*^2^ = 81.7, *p* < 0.001. In the other two conditions, we either fail to find evidence for a statistical difference across the two orders (shape-color: *χ*^2^ = 1.96, *p* = 0.376) or the difference is marginally non-significant (size-nationality: *χ*^2^ = 10.8, *p* = 0.004). If we choose a higher significance threshold, such as 0.05, the latter association would be weakly significant. However, as mentioned in the Introduction, apart from the nature of 0.05 as a convention rather than a fixed critical point, such thresholds do not offer black or white proof of evidence. In this sense, whichever threshold one assumes, the following pattern remains stable: the subjective comment-material condition stands out as showing the strongest congruent-incongruent effect, followed by the condition size-nationality, where the effect is considerably weaker, which is followed by the condition shape-color, where we fail to find evidence for the effect.

We then treated the data as ordinal. In agreement with the similarities that Endresen & Janda (2016) observed in their results when applying different statistical tests to the same pool of data, the results we obtained through ANOVAs are in agreement with those obtained through chi-squared tests. Once more, the comparison of congruent to incongruent orders reaches statistical significance in the third condition (subjective comment-material: *F* = 46.18, *p* = 0.00245), but not in the other two (shape-color: *F* = 0.766, *p* = 0.431, size-nationality: *F* = 3.097, *p* = 0.153).

**Figure 3:**
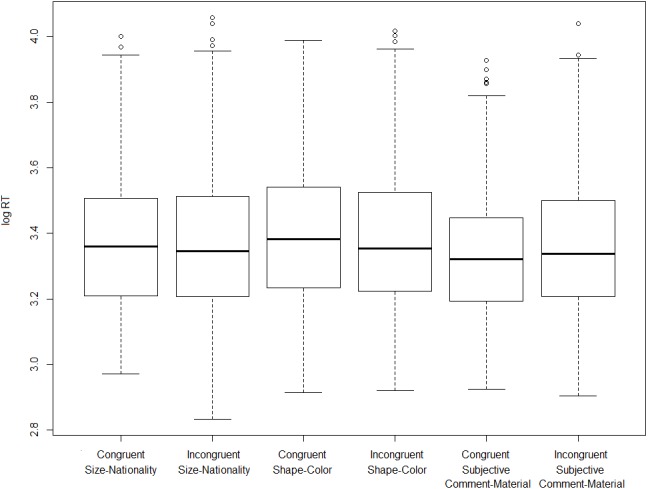
Log-transformed reaction times across orders and conditions (monolinguals).

**Figure 4:**
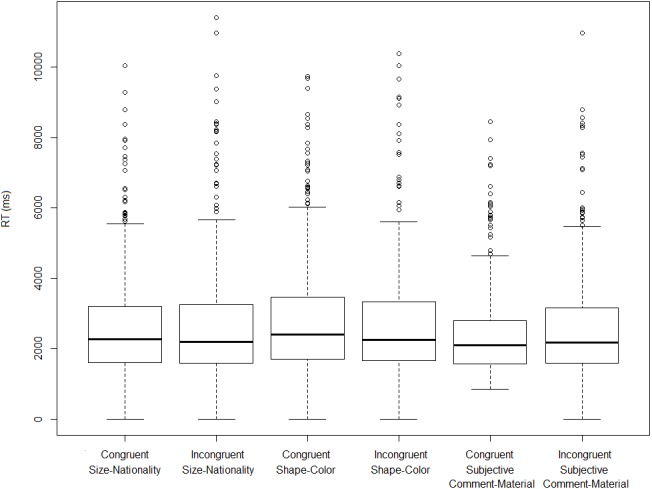
Inverse-transformed reaction times across orders and conditions (monolinguals).

Turning now to the on-line measure, on the basis of the observed reaction times we fail to establish that incongruent orders take longer to process (i.e., to assign an acceptability rating to) than congruent ones. Figures 3 and 4 show log-transformed and inverse-transformed reaction times.

**Figure 5:**
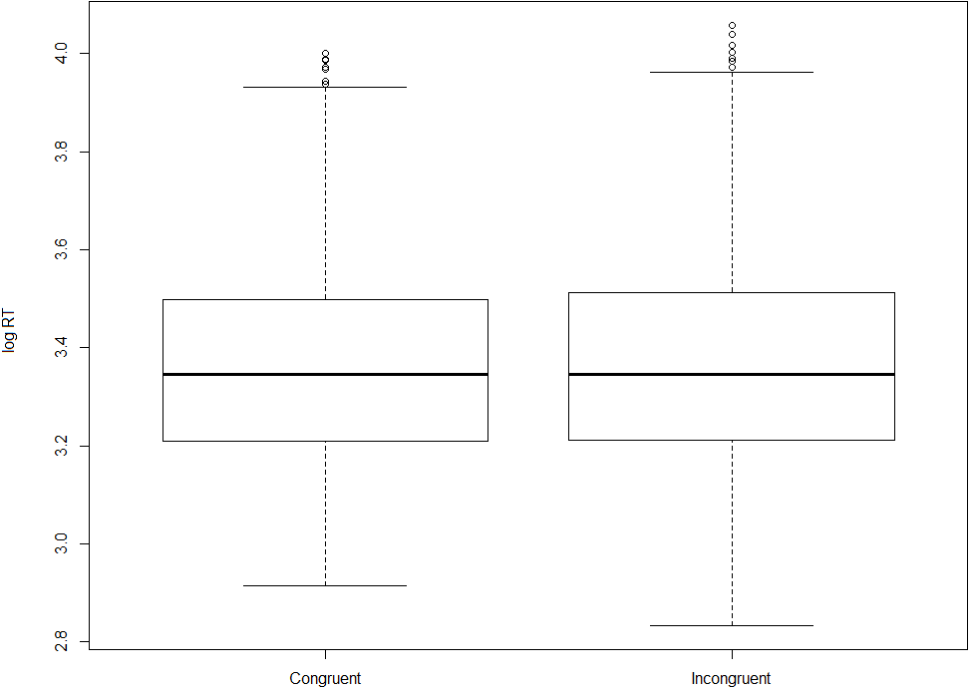
Log-transformed reaction times split by congruency (monolinguals).

**Figure 6:**
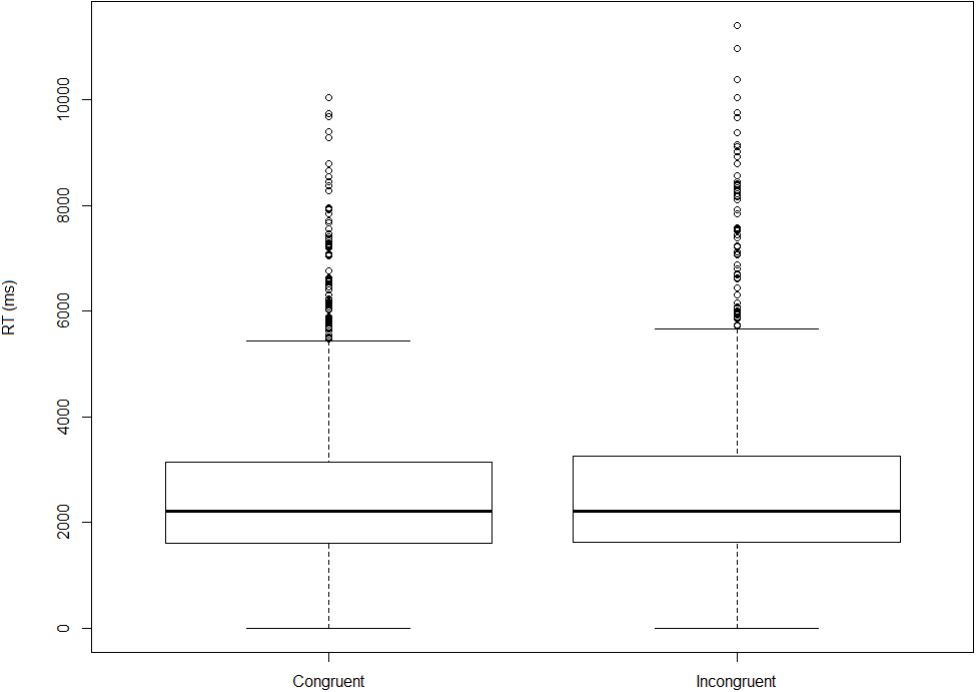
Inverse-transformed reaction times split by congruency (monolinguals).

Figures 5 and 6 present the overall log-transformed and inverse-transformed reaction times respectively, split on the basis of order (congruent vs. incongruent).

A generalized linear model with reaction times as a continuous variable showed the effect of condition to be significant (*χ*^2^ = 18.5, *p* < 0.001). We then analyzed the results separately for each condition. Taking the null hypothesis to be that there is no difference between the two orders, three paired samples t-tests, one per condition, showed that the comparison between congruent and incongruent orders does not reach significance in any condition: size-nationality: *t* = 0.108, *p* = 0.914, shape-color: *t* = 1.431, *p* = 0.155, subjective comment-material: *t* =  − 2.3825, *p* = 0.019. Even if a higher statistical threshold is set, after correcting for multiple comparisons (0.05/3), none of these differences reaches statistical significance.

The medians in Figs. 3 and 4 show the second striking result of this experiment. To explain it, let’s consider the condition size-nationality. There is a large consensus in the literature that the canonical, unmarked order (referred to here as ‘congruent order’) is the one in which the size adjective precedes the nationality adjective (see (4) and footnote 2). As mentioned already, our results show that violating this ordering constraint does not induce a statistically significant difference in terms of processing time, which should have been the case had the high acceptability been due to ‘rescuing’ the incongruent orders. Yet, had this significance threshold been reached, we wouldn’t have demonstrated that incongruent orders are timewise costlier to process than congruent ones, *but the opposite*. As shown in Figs. 3 and 4, in the condition size-nationality, it is the *congruent* order that takes on average slightly longer to process compared to the incongruent order.

**Figure 7:**
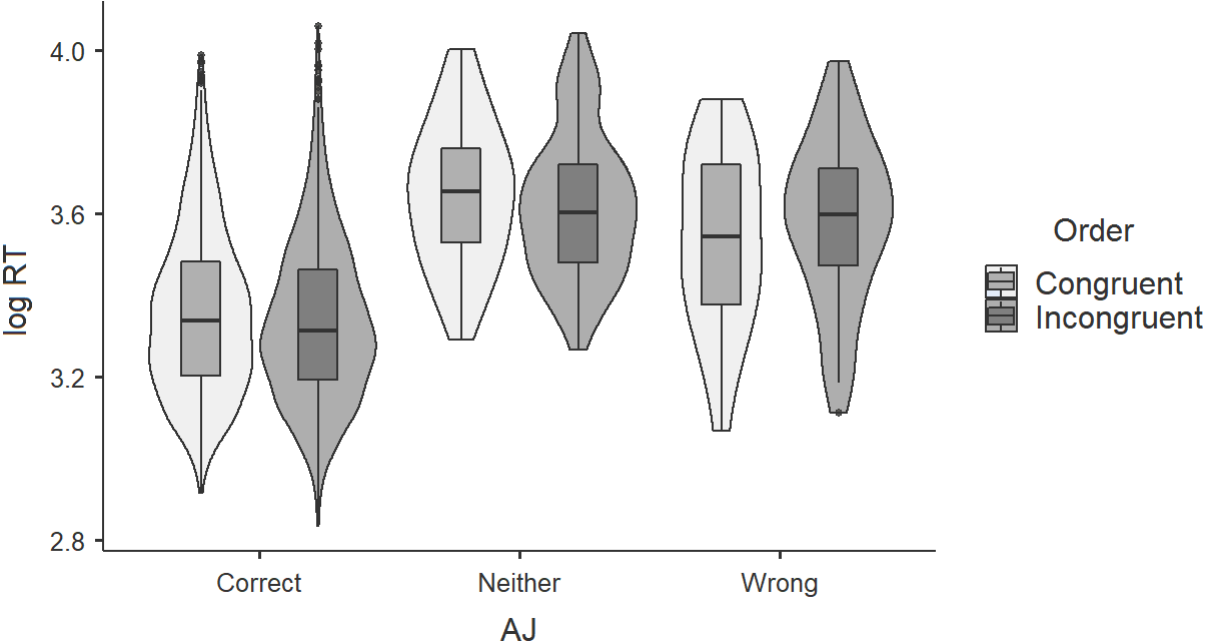
Acceptability judgments (AJ) and log-transformed reaction times (RT) for congruent and incongruent orders (monolinguals).

Figure 7 brings the assigned judgment into the picture. More specifically, it shows that incongruent orders that received the judgment ‘correct’ are not accompanied by longer reaction times, neither in comparison with their congruent counterparts that received the same judgment, nor with the other judgments in incongruent orders. If the judgment ‘correct’ was given to incongruent orders following some ‘rescuing’, the corresponding reaction times should be longer than those of their congruent counterparts, but this does not seem to be the case. Additionally, Fig. 7 shows that the reaction times look remarkably similar in both congruent and incongruent orders when split for judgment.

As the Discussion section will make clear, if the incongruent order was indeed a marked order, made possible only through some special licensing, it should have taken more time to process than the unmarked one, not less. In light of our results that find this not to be the case, one may ponder the validity and rigidity of claims and predictions that rest on (4).

## Results of Experiment 2

One of the biggest problems in science concerns replicability and reproducibility (Baker, 2015). As Ioannidis (2005) shows, a large part of published results is three things: statistically significant, underpowered, and false. We thus aimed to evaluate the results of the first experiment through a second experiment that involved the same design and methodology, but a different participant group.

In this round of testing, we recruited and tested *n* = 30 bidialectal speakers of Standard and Cypriot Greek (Table 2). All participants were Greek Cypriots who stated having Greek as their native language (see Rowe & Grohmann, 2013 for an analysis of the current sociolinguistic state of Greek-speaking Cyprus). We treated the obtained data in the same way as in experiment 1. With respect to acceptability judgment ratings, 0.18% of the data were eliminated from congruent orders and 0.09% of the data were eliminated from incongruent orders. Only automatic responses were removed. With respect to reaction times, 0.56% of the data were eliminated from congruent orders and 0.37% from incongruent orders.

**Figure 8:**
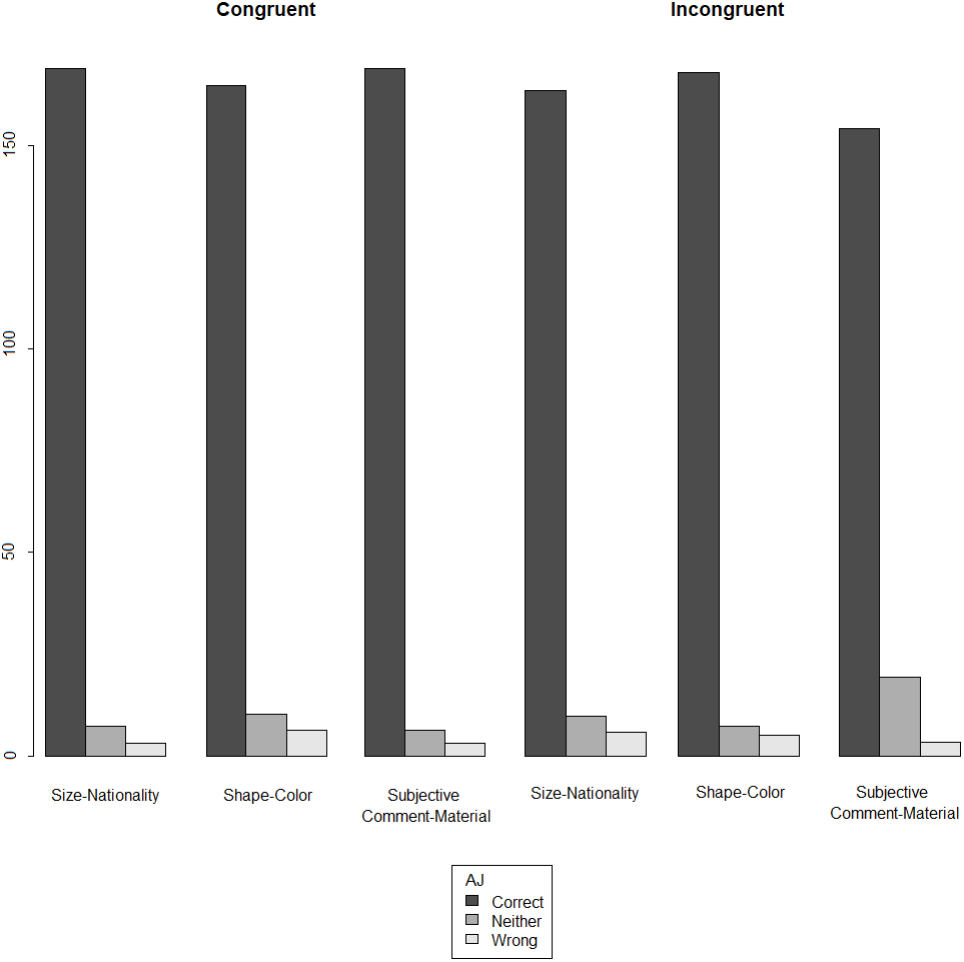
Acceptability judgment (AJ) ratings across orders and conditions in bidialectals (30 participants ×36 test items).

The results of the second experiment largely agreed with those of the first one, both for the on-line and the off-line measure. Figure 8 shows that, for both orders, in all three conditions, the judgment ‘correct’ is the predominant response. As in experiment 1, we observe a certain uniformity across the congruent and the incongruent orders in terms of the assigned ratings of acceptability: in both orders, the most frequent judgment is ‘correct’, followed by ‘neither correct nor wrong’, and ‘wrong’ following as the least attested judgment.

The judgment ‘correct’ was so pervasive in all conditions that we fail to obtain evidence for an effect of condition: treating the acceptability judgment ratings as a multinomial categorical variable, we fail to find significant evidence for such an effect using a generalized linear model (*χ*^2^ = 3.57, *p* = 0.468). Treating the reaction times as a linear continuous variable, again we do not observe a significant effect of condition (*χ*^2^ = 3.35, *p* = 0.188). Given that condition was not found to be significant in either measure, we examined the effect of order in the overall sample too, collapsing the three conditions. We fail to find evidence for a significant difference in the treatment of the congruent vs. incongruent orders, both as regards the acceptability judgment ratings (*χ*^2^ = 3.89, *p* = 0.143) and the reaction times (*χ*^2^ = 3.48, *p* = 0.062).

Aiming to establish comparisons across the two experiments, we split for condition and ran three chi-squared tests, comparing acceptability ratings in the congruent and incongruent orders. At a confidence level of 99%, *α* = 0.0033 after Bonferroni correction for multiple comparisons. Unlike the first experiment, where the null hypothesis was rejected in the condition subjective comment-material, in this experiment the difference between the ratings assigned to the congruent orders and incongruent orders does not reach statistical significance for any condition: size-nationality: *χ*^2^ = 1.6, *p* = 0.449, shape-color: *χ*^2^ = 0.645, *p* = 0.724, subjective comment-material: *χ*^2^ = 8.28, *p* = 0.016. If we choose a higher significance threshold, such as 0.05, *a* = 0.016 after correcting for multiple comparisons, the difference in the last condition would marginally be weakly significant. Importantly, the overall pattern that was observed in the first experiment is also found in the dataset from the second experiment: the subjective comment-material condition shows the strongest congruent-incongruent effect, followed by the condition size-nationality, where the effect is weaker, followed by the condition shape-color, where we fail to find evidence for the effect.

In the second analysis of the acceptability judgment ratings, the data were treated as ordinal in the way presented above for experiment 1. Once more, the results corroborate those of the first experiment, as the third condition, subjective comment-material, shows the strongest congruent-incongruent effect: *F* = 2.11, *p* = 0.177, followed by size-nationality: *F* = 0.672, *p* = 0.432, and then by shape-color: *F* = 0.0588, *p* = 0.813. Unlike experiment 1, where the difference in the third condition was weakly significant, none of the differences reached significance in experiment 2.

**Figure 9:**
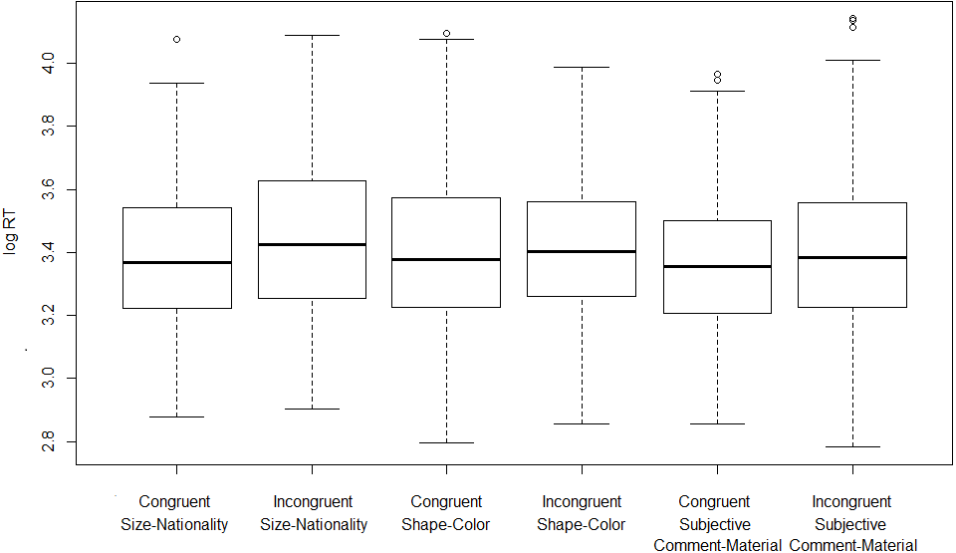
Log-transformed reaction times across orders and conditions (bidialectals).

In relation to the reaction times, Fig. 9 shows that incongruent orders took approximately as long as the congruent ones to get assigned an acceptability rating, similar to what was found in experiment 1. As in the case of the latter, three paired samples *t*-tests, one per condition, showed that the comparison between congruent and incongruent orders does not reach significance in any condition: size-nationality: *t* =  − 1.58, *p* = 0.124, shape-color: *t* =  − 0.536, *p* = 0.596, subjective comment-material: *t* =  − 2.36, *p* = 0.025. If a higher statistical threshold is assumed, after correcting for multiple comparisons, none of the differences between the reaction times in congruent vs. incongruent stimuli would be statistically significant.

## Discussion

The two research questions behind this project are the following:

(QI) Is there a universal hierarchy for adjective ordering that—in the absence of any special context that lifts restrictions—gives rise to an unmarked order?

(QII) Do ordering restrictions, to the degree they are attested, correspond to hard constraints or to mere preferences?

It is perhaps useful to start the discussion of these questions by pointing out a common misconception. Some linguists have at times been critical of the notion of Universal Grammar, invoking in their arguments an (erroneous) equation of the latter with universality. However, being universal is not synonymous with being encoded in Universal Grammar, and “parts of the human mind/body that are not specifically linguistic may obviously be universal” (Sigurðsson, 2012: 327). This is important in relation to the claim we want to pursue in two ways.

First, universal preferences might be the outcome of general cognitive principles, not specific to linguistics (i.e., Chomsky’s 2005 ‘third factor’ in language design). Through endorsing this idea and adducing empirical evidence for it, we pave the way for a deflationist approach to linguistic cognition, by seeking to ascribe to Universal Grammar (i.e., Chomsky’s 2005 ‘first factor’) as little specificity as possible: Our results are at odds with the idea that there is an innately wired hierarchy in Universal Grammar that constrains adjective ordering. We thus concur with Kotowski & Härtl (2019) on the untenability of the cartographic conception of the origin of such constraints being a syntactic hierarchy encoded in Universal Grammar: consider, for instance, the claim in Cinque & Rizzi (2008) on how “comparison of many different languages may provide evidence for determining the precise relative order of the different functional projections by combining the partial orders overtly manifested by different languages into what, in principle, *should be a unique consistent order/hierarchy, imposed by UG*” (p. 48, emphasis added). However, we part ways with Kotowski & Härtl (2019) on the topic of the very existence of such constraints. As mentioned in the Introduction, Kotowski & Härtl’s (2019) results do show the presence of one hard constraint: relational adjectives (e.g., adjectives denoting nationality or material) are always found closer to the noun than other adjectives. Figures 1 and 8, however, show that the participants of our experiments largely accepted as ‘correct’ the incongruent order of the size-nationality condition, which had the size adjective closer to the noun. In other words, our results suggest that not only is there reason to doubt that a Universal Grammar-encoded hierarchy places a hard constraint on adjective ordering, but also to doubt that such a hard constraint exists *at all*, regardless of its origin. One might argue in favor of a mere preference instead of a hard constraint, but even this is debatable in one of the conditions we tested; compare, for instance, the congruent and incongruent order in the condition shape-color in Fig. 1. With respect to the origin of such preferences, they are likely formed on the basis of the notions that the cognitive approaches have been investigating (i.e., inherentness, subjectivity, absoluteness), as it is implausible that linguistic communities that speak typologically different languages would have somehow settled on similar orderings in the absence of a shared cognitive basis. The way these preferences are externalized and synchronically attested within a linguistic community is additionally affected by a pragmatic happenstance. In the context of the latter, it is possible that the increase in the judgments ‘neither correct nor wrong’ and ‘wrong’ in the incongruent orders reflects a subconscious knowledge of what is statistically more frequent in the input, which in turn is based on what the grammar books define as the norm (see also Kotowski & Härtl, 2019; Wulff, 2003; Champollion, 2006 on pattern conventionalization). Despite this increase, it is abundantly clear that our participants’ predominant evaluation is ‘correct’ both in the congruent and the incongruent orders across conditions.

Our participants almost categorically assigned the judgment ‘wrong’ to the ungrammatical fillers of the task, so there is no doubt that they *can* recognize an ungrammatical sentence when they encounter one. Also, one would be wrong to think that this high acceptance of the incongruent orders as ‘correct’ is due to the fact that participants were interpreting these orders in comparison to the ungrammatical fillers—in the sense that one could have judged the fillers as more markedly ill-formed, something that could have led to some favorability in the judgment of the incongruent orders—for at least four reasons.

First, if that was the case, the predominant answer in incongruent orders should be ‘neither correct nor wrong’, because research on Likert scales has shown that when a middle, ‘neither’ option is included as a possible response option, more than a fifth of the sample shifts to it, especially when some ambivalence is involved (Schuman & Presser, 1996; Baka, Figgou & Triga, 2012; Nadler, Weston & Voyles, 2015).

Second, as mentioned in Materials & Methods, all ungrammatical fillers were ungrammatical precisely due to violations of ordering constraints, and they were successfully recognized as such by the participants. It seems straightforward that if an adjective ordering constraint truly existed in our participants’ linguistic repertoire, the effects of its violation should show up the way they did for the ordering constraints violated in ungrammatical fillers.

Third, in the design and piloting phases of this experiment, the congruent and incongruent orders were tested separately and without any fillers. Again, the results showed a large acceptance of the incongruent orders as ‘correct’. In line with standard practice (Peat et al., 2002), the results obtained from the piloting phase were not included in the above reports and analyses. It is also worth highlighting that upon the completion of the first experiment, 11 participants who assigned the rating ‘correct’ to all incongruent orders were asked whether they find any interpretive difference between ‘a small Chinese mobile’ or ‘a Chinese small mobile’ (i.e., a combination that was tested in the experiment). The answer was negative in all cases.

**Figure 10:**
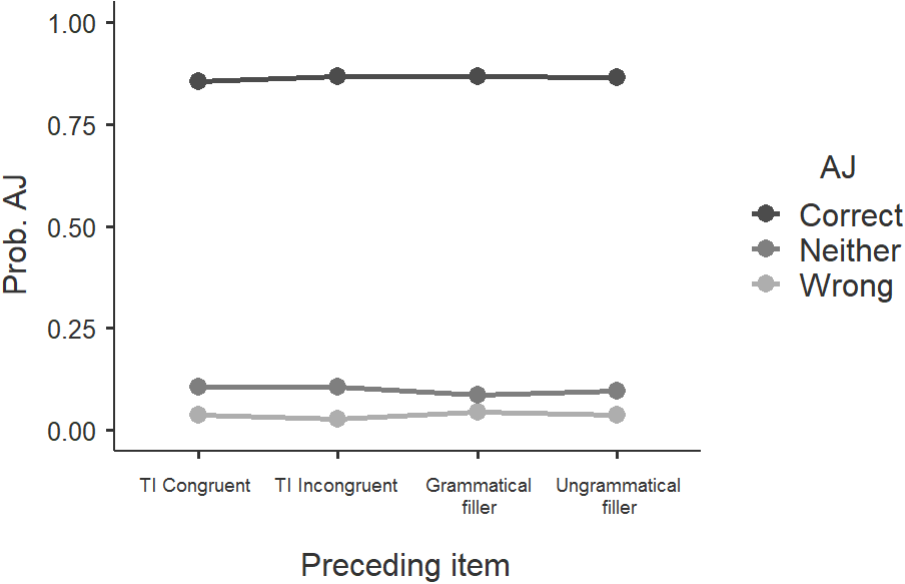
The effect of preceding item on the acceptability judgment (AJ) ratings of incongruent orders (monolinguals, experiment 1). TI: Test Item, Prob.: Probability.

Fourth, priming effects are predicated on the notion of grammaticality, in the sense that such extra-grammatical influence targets grammatical sentences, but not ungrammatical ones (Sprouse, 2007). If our incongruent orders were ungrammatical in this out-of-context presentation of the stimuli, they should be unaffected by extra-grammatical effects induced by preceding items. If, on the other hand, the incongruent orders were grammatical but non-canonical and hence “rescued” under special licensing conditions, the reaction times should have reflected this, and they did not. Last, if they were falling into the grey area of sentences that are neither clearly grammatical nor clearly ungrammatical (Sprouse, 2007), given the presence of both grammatical and ungrammatical fillers that could push judgments to opposite directions, the middle option on the Likert scale should have been the predominant answer, and it was not. With respect to partial/moderate grammaticality, some studies have found that repeated exposure facilitates acceptability (Luka & Barsalou, 2005; Hofmeister, Staum Casasanto & Sag, 2013), but given the mixed results in the literature on this topic, it has been argued that “the field lacks a firm understanding of why some structures get better with exposure and not others” (Hofmeister, Staum Casasanto & Sag, 2013: 53). When it comes to our results, the incongruent orders in our experiments were not judged as acceptable only after repeated exposure and/or only when being encountered right after ungrammatical fillers. Instead, they were judged as acceptable the first time they were encountered, regardless of whether they were preceded by (i) a grammatical filler, (ii) an ungrammatical filler, or (iii) a test item. To illustrate this in the context of experiment 1, Fig. 10 shows all the acceptability judgments assigned to test items in the incongruent order in relation to the item they were preceded by. There are four options with respect to the latter: a test item from the congruent order, a test item from the incongruent order, a grammatical filler or an ungrammatical filler. Figure 10 suggests that there is no peak in the acceptability of incongruent orders when they appear after an ungrammatical filler, hence the above-mentioned hypothesis about potential favorability seems uncorroborated. A generalized linear model analysis also fails to reveal an effect of ‘preceding item’ (*χ*^2^ = 1.42, *p* = 0.965).

For these four reasons together, we conclude that the high acceptability of the incongruent orders attests to their status as grammatical sentences.

At the same time, the results of both experiments seem to map onto the predictions made by (4), revealing a ‘distance’ effect: The strongest congruent-incongruent effect is found for the condition subjective comment-material, which involves two adjectives that lie at the two edges of (4). This effect is followed by a considerably weaker effect for the condition size-nationality, which features adjectives that are separated by a number of nodes in (4), and a yet weaker effect for the condition shape-color, the adjectives of which are adjacent on the hierarchy. This pattern of effects across conditions is in line with what Adam & Schecker (2011) find for ERP data on German adjective ordering and with the preferences Scontras, Degen & Goodman (2017) obtained for English. In other words, the two classes farthest apart in the hierarchy result in the strongest effect (which, however, is *not* statistically significant in the second experiment, although still stronger than that of the other two conditions), while the other two classes show a much weaker effect that ranges from insignificant to moderate, depending on the statistical test and threshold one employs. This ‘distance’ effect does not validate the hierarchy in (4), because the effect is compatible also with proposals that derive ordering preferences on the basis of cognitive notions such as subjectivity (Scontras, Degen & Goodman, 2017): In ‘beautiful woolen dress’, judgments about the subjective comment would likely be more variable than judgments about material, and the difference in terms of subjectivity among the adjectives that make up this adjective pair is greater than the one found in the adjective pairs of the other two conditions. Moreover, given that all judgments were eventually attested in all incongruent orders, this flexibility is concomitant with theories that are based on cognitive/psychological constructs rather than innately encoded—and as such *inflexible*—hierarchies such as the one in (4).

The second reason that makes Sigurðsson (2012) claim important for the purposes of this discussion has to do with defining the target of our claims. We do not put an argument against Universal Grammar itself, but against the idea that ordering constraints for adjective placement are encoded there or derive from hierarchies or other primitives encoded there. There may be preferences attested in some languages and to some degrees, but our results suggest that there is no such thing as a single, universal, canonical/unmarked order. Instrumental in supporting the latter claim are the reaction times we obtained. In our experiment, there is no statistically significant difference between the time needed to successfully compute the congruent vs. the incongruent orders. As a matter of fact, some canonical orders even took slightly longer to process than their incongruent counterparts. Yet, a long line of research in both linguistics and psychology has affirmed that unmarked sentences that do not involve complex syntactic operations are processed faster (Rösler et al., 1998; Erdocia et al., 2009; Tamaoka, Kanduboda & Sakai, 2011). Given that it has long been argued that reaction times are sensitive to the number and type of the operations being executed and to the number of alternative interpretations that must be computed (Bates et al., 1982), our inability to find a statistically significant difference between the time needed to process the two types of orders, congruent and incongruent, casts some doubt on the idea that a single, universal, canonical/unmarked order exists. (QI) thus receives a negative answer, while the answer to (QII) seems to be that ordering restrictions, *to the degree they are attested*, reflect mere preferences. Our results are indirectly related to (QII) in the following way. If violations of the hierarchy do not give rise to robust judgments of ungrammaticality, it is unlikely that one can sustain the cartographic idea of an innate hierarchy anymore. Our results suggest that adjective ordering is based on preferences (QII) that do not seem to have a syntactic basis—and they do not seem to have a syntactic basis because violating the alleged syntactic hierarchy does not lead our participants to judge these sentences as ungrammatical. That is already very different from the claim that Universal Grammar (or any other version of innateness) expresses the way elements are organized via *universal syntactic hierarchies*, which is the claim pursued in cartography (see, for instance, Cinque & Rizzi, 2008: 53 and Panayidou, 2013: 11). The observed preferences are stronger in some conditions than others; something that may boil down to the ‘distance’ effect: the farther apart two adjectives are in terms of intervening nodes in (4), the stronger the preference for the congruent order.

The general take-home message that emerges from this experiment concerns approaches that ascribe to our innate endowment for language a richness of hierarchies and constraints. It has been suggested that an overspecified conception of Universal Grammar entails a certain degree of biological implausibility (Boeckx & Leivada, 2014), inevitably making arguments and theories that rely on this conception very hard to defend from an interdisciplinary point of view. The key contribution of the present research is the demonstration that some of the predictions made by approaches that postulate a rich Universal Grammar do not seem to be borne out experimentally.

## Conclusions

This research put the nature and rigidity of linguistic hierarchies to test, taking multiple adjective placement as a case study. We developed an on-line forced choice experiment that measured (i) acceptability judgment ratings and (ii) reaction times, in a sample of monolingual, neurotypical, adult speakers of Standard Greek (*n* = 140) and a smaller sample of bidialectal, adult speakers of Standard and Cypriot Greek (*n* = 30). The tasks were designed to compare what happens when people are asked to process sentences that either comply with or violate allegedly universal ordering constraints that have been described as the outcome of innately wired hierarchies. Three striking findings emerged. First, hierarchy-compliant (i.e., congruent) orders were treated quite similar to hierarchy-incompliant (i.e., incongruent) ones, in the sense that the two did not differ in terms of the assigned ratings of acceptability in two out of three tested conditions: Participants generally judged both types of orders as ‘correct’. Second, in the analysis of the obtained reaction times we failed to find evidence that incongruent orders took longer to process, in stark contrast to what the theory on processing marked vs. unmarked orders predicts. Third, the three conditions we tested produced results that differed with respect to the acceptability of the incongruent orders in a way that revealed a ‘distance’ effect: The farther apart two adjectives classes are along the lines of the proposed hierarchy (4), the bigger the difference between congruent and incongruent orders in terms of acceptability. This difference may or may not reach statistical significance, depending on the statistical threshold and the statistical test one employs.

Taking these findings together, we have argued that there is no universal hierarchy for adjective ordering imposing a hard constraint which then translates into one rigid, unmarked order. Turning away from the notion of a hard constraint, it is likely that future research on this topic will shed light on the nature and strength of the attested ordering preferences, hopefully in a way that can hold up to interdisciplinary scrutiny.

##  Supplemental Information

10.7717/peerj.7438/supp-1 Supplemental Information 1 Reading key

10.7717/peerj.7438/supp-2 Supplemental Information 2 Monolinguals - experiment 1

10.7717/peerj.7438/supp-3 Supplemental Information 3 Bidialectals (experiment 2)

10.7717/peerj.7438/supp-4 Supplemental Information 4 Test items for bidialectals (experiment 2)

10.7717/peerj.7438/supp-5 Supplemental Information 5 Test items for monolinguals (experiment 1)

## Additional Information and Declarations

### Data Availability

The following information was supplied regarding data availability: The raw data are available in the Supplemental Files and at Leivada, Evelina; Westergaard, Marit, 2019, “Replication Data for: Universal linguistic hierarchies are not innately wired”, https://doi.org/10.18710/NTLLUF, DataverseNO, V1.
