# Supplementary material for: Universal linguistic hierarchies are not innately wired. Evidence from multiple adjectives
Source: PeerJ. 2019 Aug 1;7:e7438. doi: 10.7717/peerj.7438 (PMC6679903; doi:10.7717/peerj.7438)
Supplement: Supplemental Information 4 [file peerj-07-7438-s004.pdf]

Design features

- One verb per block: *get, see, and find* in 1SG past tense, all two syllables long
- All objects are full DPs-Spelke objects (3 syllables), no repetitions
- All Adjective Phrases appear in direct object position (a combination of two adjectives, 6 syllables in total), no repetitions
- 6 items per condition x 3 conditions (i. size > nationality/origin, ii. shape > color, iii. subjective comment > material). Each condition features two orders (size before/after nationality, shape before/after color, subjective comment before/after material)
- Ratio fillers – test structures 2:1
- Bidialectals were tested in two varieties: Standard Greek and Cypriot Greek. Test items in Standard Greek are given in the form 'Test items for monolinguals'. Test items in Cypriot Greek are given below.

// Size, nationality/origin

1. Έπια ένα μιτσή κυπριακό χαλλούμι.
(Condition 1, order: size before nationality) – I bought a small Cypriot halloumi.

2. Έπια ένα φαρδύ εγγλέζικο σεντόνι.
(Condition 1, order: size before nationality) – I bought a wide English bedsheet.

3. Έπια ένα στενό ισπανικό βρασιόλι.
(Condition 1, order: size before nationality) – I bought a narrow Spanish bracelet.

4. Έπια ένα ελληνικό πασσιή σακκούι.
(Condition 1, order: nationality before size) – I bought a heavy Greek jacket.

5. Έπια ένα κινέζικο μιάλο τηάνι.
(Condition 1, order: nationality before size) – I bought a big Chinese pan.

6. Έπια ένα βουλγάρικο λεπτό τσεντούι.
(Condition 1, order: nationality before size) – I bought a thin Bulgarian purse.

// Shape, color

7. Είδα ένα μακρύ πορτοκκαλί κολότζι.
(Condition 2, order: shape before color) – I saw a long orange zucchini.

8. Είδα ένα στροτζυλό πράσινο κουττούι.
(Condition 2, order: shape before color) – I saw a round green little box.

9. Είδα ένα τετράγωνο άσπρο σβηστήρι.
(Condition 2, order: shape before color) – I saw a square white eraser.

10. Είδα ένα λιλά ασύμμετρο καππέλο.
(Condition 2, order: color before shape) – I saw an asymmetric lilac hat.

11. Είδα ένα τζίτρινο μακρουλό πιπόνι.
(Condition 2, order: color before shape) – I saw a long yellow melon.

12. Είδα ένα μαυρόασπρο οβάλ πιατούι.
(Condition 2, order: color before shape) – I saw an oval black and white little dish.

// Subjective comment, material

13. Ήβρα ένα τέλειο γυάλενο φεντζιάνι.
(Condition 3, order: subjective comment before material) – I found a cool glass mug.

14. Ήβρα ένα άχρηστο ξύλενο καρεκλί.
(Condition 3, order: subjective comment before material) – I found a useless wooden chair.

15. Ήβρα ένα όμορφο χάλκινο καζάνι.
(Condition 3, order: subjective comment before material) – I found a beautiful copper cooking pot.

16. Ήβρα ένα ρούχενο άσσιημο παπούτσι.
(Condition 3, order: material before subjective comment) – I found an ugly linen shoe.

17. Ήβρα ένα μάλλενο ακριβό ριχτάρι.
(Condition 3, order: material before subjective comment) – I found an expensive woollen drape.

18. Ήβρα ένα μπρούτζενο άνετο κρεβάτι.
(Condition 6, order: material before subjective comment) – I found a comfortable brass bed.

Acceptable Fillers

19. Η Μάρω δουλεύκει στο εθνικό μουσείο.
(Maro works in the national museum)

20. Η φωνή της ακούετουν πολλά δυνατά.
(Her voice was heard very loudly)

21. Η Έλενα εν που το χωρκό του παπά μου.
(Elena is from my dad’s village)

22. Ήβραμεν ένα γκρίζο καττούι στον δρόμο.
(We found a gray kitten in the street)

23. Εξίασα το βιβλίο μου στο γραφείο.
(I forgot my book in the office)

24. Η κορούα εχόρεψε με τες φίλες της.
(The girl danced with her friends)

25. Εστράφηκεν που το ταξίδι ποσταμένη.

(She came back from the trip tired)

26. Η Σκεύη εφοήθηκεν που τες πουμπουρκές.
(Skevi got scared by the thunders)

27. Ο Αλέξανδρος επότισεν τα δεντρούθκια.
(Alexander watered the little trees)

Unacceptable Fillers

28. Εγοράσαμεν όμορφο σπιτούι ένα.
(misplaced indefinite article– intended meaning: We bought a beautiful little house)

29. Εφάμεν όρνιθα πατατούες πάλε με.
(misplaced preposition – intended meaning: We ate chicken with potatoes again)

30. Είδα τη προψές μιτσιά κόρη του Αντώνη.
(misplaced determiner – intended meaning: I saw the youngest daughter of Adonis two nights ago)

31. Νικολέττα ήβρεν καλύτερη δουλειά η.
(misplaced determiner – intended meaning: Nikoleta found a better job)

32. Το άρθρο μας εκδόθηκεν Παρασκευή την.
(misplaced determiner – intended meaning: Our article was published on Friday)

33. Ο μιτσής έφαεν τα ούλα κεραστικά.
(misplaced determiner – intended meaning: The little boy ate all the desserts)

34. Το στο τραγούδι της ακούστηκεν ράδιο.
(misplaced determiner and preposition – intended meaning: Her song was heard on the radio)

35. Χρυστάλλα η εγόρασεν ένα κινητό.
(misplaced determiner– intended meaning: Chrystalla bought a mobile)

36. Πανίκκος έκοψεν ο μερικά λουλούδια.
(misplaced determiner– intended meaning: Panicos picked up some flowers)

37. Στην ο Νικόλας εταξίδεψεν Αγγλία.
(misplaced determiner and preposition – intended meaning: Nicolas travelled to England)

38. Ήβραμεν στην ένα πεζουνούι αυλή μας.
(misplaced determiner and preposition – intended meaning: We found a little pigeon in our courtyard)

39. Η τες Αγγελική έκαμεν χαλλουμωτές.
(misplaced determiner– intended meaning: Angeliki made the halloumi pies)

40. Την Τρίτη επήαμεν ούλοι θέατρο στο.

(misplaced determiner and preposition– intended meaning: On Tuesday we all went to the theatre)

41. Έπια μεταχειρισμένο αρμάρι ένα.
(misplaced indefinite article– intended meaning: I bought a second-hand wardrobe)

42. Έκοψα τέσσερις που τον τουλίππες κήπο.
(misplaced determiner and preposition– intended meaning: I cut four tulips from the garden)

43. Έβαψα το σκαλούι της αυλής άσπρο μας.
(misplaced possessive– intended meaning: I painted the step of our courtyard white)

44. Έφα ένα κότσινο το μηλούι πρωί.
(misplaced determiner– intended meaning: I ate a red apple in the morning)

45. Έλυσα ασκήσεις με πολλή προσοχή τες.
(misplaced determiner– intended meaning: I solved the exercises with great attention)

46. Επήαμεν έναν περίπατο πόλη στην.
(misplaced determiner and preposition – intended meaning: We went for a walk in the city)

47. Με επισκέφτηκεν ανιψιά η μου προχτές.
(misplaced determiner– intended meaning: My cousin visited me two days ago)

48. Οι μαθητές επήαν εκδρομή Πάφο στην.
(misplaced determiner and preposition – intended meaning: The students went to an excursion in Pafos)

49. Νίκος ο πάει θάλασσα κάθε εφτομάν.
(misplaced determiner– intended meaning: Nick goes to the sea every week)

50. Άννα έφαεν θκυο η ελιωτές σήμερα.
(misplaced determiner– intended meaning: Anna ate two olive pies today)

51. Τα θκυο κοπελλούθκια παίζουσιν σαλόνι στο.
(misplaced determiner and preposition – intended meaning: The two kids play in the living room)

52. Το μωρούι έπαιζεν παιχνίδι με το του.
(misplaced determiner and preposition – intended meaning: The baby was playing with its toy)

53. Το γραφείο εν μου γεμάτο τετράδια.
(misplaced possessive– intended meaning: My office is full of notebooks)

54. Μαρίνος ο εστράφηην έσσω πιττωμένος.
(misplaced determiner– intended meaning: Marinos returned home drunk)
