# Supplementary material for: Universal linguistic hierarchies are not innately wired. Evidence from multiple adjectives
Source: PeerJ. 2019 Aug 1;7:e7438. doi: 10.7717/peerj.7438 (PMC6679903; doi:10.7717/peerj.7438)
Supplement: Supplemental Information 5 [file peerj-07-7438-s005.pdf]

Design features

- One verb per block: *get, see, and buy* in 1SG past tense, all two syllables long
- All objects are full DPs-Spelke objects (3 syllables), no repetitions
- All Adjective Phrases appear in direct object position (a combination of two adjectives, 6 syllables in total), no repetitions
- 6 items per condition x 3 conditions (i. size > nationality/origin, ii. shape > color, iii. subjective comment > material). Each condition features two orders (size before/after nationality, shape before/after color, subjective comment before/after material)
- Ratio fillers – test structures 2:1
- Ratio acceptable stimuli – unacceptable stimuli 1:1
- Lemma frequency >1 for all content words in all test structures

Test Items

// Size, nationality/origin

1. Βρήκα μία μεγάλη γαλλική βεντάλια.
(Condition 1, order: size before nationality) – I found a big French fan.

2. Βρήκα ένα μικρό γερμανικό κινητό.
(Condition 1, order: size before nationality) – I found a small German mobile.

3. Βρήκα ένα λεπτό ελβετικό ρολόι.
(Condition 1, order: size before nationality) – I found a thin Swiss watch.

4. Βρήκα ένα ιταλικό μακρύ φόρεμα.
(Condition 1, order: nationality before size) – I found an Italian long dress.

5. Βρήκα ένα κινέζικο χοντρό πάπλωμα.
(Condition 1, order: nationality before size) – I found a Chinese fat duvet.

6. Βρήκα ένα σουηδικό φαρδύ κρεβάτι.
(Condition 1, order: nationality before size) – I found a Swedish wide bed.

// Shape, color

7. Είδα ένα τετράγωνο μαύρο τραπέζι.
(Condition 2, order: shape before color) – I saw a square black table.

8. Είδα μία μακρόστενη γκρίζα κουβέρτα.
(Condition 2, order: shape before color) – I saw a rectangular gray blanket.

9. Είδα έναν οβάλ ασπρόμαυρο καναπέ.
(Condition 2, order: shape before color) – I saw an oval black-white sofa.

10. Είδα ένα καφέ τριγωνικό μαντίλι.
(Condition 2, order: color before shape) – I saw a brown triangular scarf.

11. Είδα έναν άσπρο κυλινδρικό σωλήνα.
(Condition 2 order: color before shape) – I saw a white cylindrical tube.

12. Είδα ένα κίτρινο στρογγυλό διαμάντι.
(Condition 2, order: color before shape) – I saw a yellow round diamond.

// Subjective comment, material

13. Πήρα ένα κομψό μεταλλικό μπαούλο.
(Condition 3, order: subjective comment before material) – I got an elegant metal trunk.

14. Πήρα μία υπέροχη χρυσή καρφίτσα.
(Condition 3, order: subjective comment before material) – I got an awesome gold brooch.

15. Πήρα ένα φρικτό δερμάτινο καπέλο.
(Condition 3, order: subjective comment before material) – I got a horrible leather hat.

16. Πήρα ένα ξύλινο ωραίο γραφείο.
(Condition 3, order: material before subjective comment) – I got a wooden nice desk.

17. Πήρα ένα βαμβακερό απλό σεντόνι.
(Condition 3, order: material before subjective comment) – I got a cotton plain bedsheet.

18. Πήρα ένα μάλλινο όμορφο φουστάνι.
(Condition 3, order: material before subjective comment) – I got a woolen beautiful dress.

Acceptable Fillers

19. Η Λίνα δουλεύει στο εθνικό μουσείο.
(Lina works in the national museum)

20. Η τηλεόραση παίζει πολύ δυνατά.
(The television is playing very loudly)

21. Η Βίκυ είναι η καλύτερή μου φίλη.
(Vicky is my best friend)

22. Πούλησα το καινούργιο αυτοκίνητό μου.
(I sold my new car)

23. Ξέχασα το τετράδιο στο δωμάτιο.
(I forgot the notebook in the room)

24. Είπα ένα ποίημα στη σχολική γιορτή.
(I said a poem in the school celebration)

25. Γύρισε από το ταξίδι κουρασμένος.
(He came back from the trip tired)

26. Το κορίτσι φοβήθηκε από τις βροντές.
(The girl got scared by the thunders)

27. Ο Αλέξης περιποιήθηκε τον κήπο.
(Alexis took care of the garden)

Unacceptable Fillers

28. Αγοράσαμε όμορφο σπιτάκι ένα.
(misplaced indefinite article– intended meaning: We bought a beautiful little house)

29. Φάγαμε ένα ωραίο ταβερνάκι σε.
(misplaced preposition – intended meaning: We ate in a nice little tavern)

30. Είδα το προχθές μικρό παιδί του Αντώνη.
(misplaced determiner – intended meaning: I saw Antoni's youngest kid two days ago)

31. Αλεξάνδρα βρήκε καινούργια δουλειά η.
(misplaced determiner – intended meaning: Alexandra found a new job)

32. Το άρθρο δημοσιεύτηκε Σάββατο το.
(misplaced determiner – intended meaning: The article was published on Saturday)

33. Το αγοράκι έφαγε τα όλα γλυκά.
(misplaced determiner – intended meaning: The little boy ate all the desserts)

34. Το στο τραγούδι ακούστηκε ραδιόφωνο.
(misplaced determiner and preposition – intended meaning: The song was heard on the radio)

35. Γείτονες οι αγόρασαν καινούργιο σπίτι.
(misplaced determiner– intended meaning: The neighbours bought a new house)

36. Μαρίνα μάζεψε η κόκκινα λουλούδια.
(misplaced determiner– intended meaning: Marina picked up red flowers)

37. Στην ο Νικόλας ταξίδεψε Αμερική.
(misplaced determiner and preposition – intended meaning: Nicolas travelled to America)

38. Βρήκα στην ένα μικρό σκυλάκι αυλή μου.
(misplaced determiner and preposition – intended meaning: I found a little dog in my courtyard)

39. Η τα Ειρήνη έβαψε μαλλιά της ξανθά.
(misplaced determiner– intended meaning: Irene dyed her hair blonde)

40. Χτες βράδυ πήγαμε κινηματογράφο στον.

(misplaced determiner and preposition– intended meaning: Last night we went to the cinema)

41. Πήρα μεταχειρισμένο αμάξι ένα.
(misplaced indefinite article– intended meaning: I got a used car)

42. Είδα τέσσερα στον τριαντάφυλλα κήπο.
(misplaced determiner and preposition– intended meaning: I saw four roses in the garden)

43. Έβαψα την πόρτα της αυλής πράσινη μας.
(, misplaced possessive– intended meaning: I painted the door of our courtyard green)

44. Έφαγα ένα κόκκινο το μήλο πρωί.
(misplaced determiner– intended meaning: I ate a red apple in the morning)

45. Έλυσα ασκήσεις με πολλή προσοχή τις.
(misplaced determiner– intended meaning: I solved the exercises with great attention)

46. Πήγα για ποτό αγαπημένο στο μου μπαρ.
(misplaced determiner and preposition – intended meaning: I went for a drink to my favorite bar)

47. Προχθές με επισκέφτηκαν ξαδέρφια τα μου.
(misplaced determiner– intended meaning: My cousins visited me two days ago)

48. Οι μαθητές έγραψαν χθες διαγώνισμα μας.
(misplaced possessive– intended meaning: Our students had an exam yesterday)

49. Ηλίας ο κολυμπάει κάθε βδομάδα.
(misplaced determiner– intended meaning: Elias swims every week)

50. Μίνα τρώει σοκολάτα η κάθε μέρα.
(misplaced determiner– intended meaning: Mina eats chocolate every day)

51. Τα παιδιά παίζουν χαρούμενα σαλόνι στο.
(misplaced determiner and preposition – intended meaning: The children are playing happily in the living room)

52. Το μωρό παίζει ήσυχα κούκλα με την του.
(misplaced determiner and preposition – intended meaning: The baby plays quietly with her doll)

53. Το γραφείο είναι μου γεμάτο βιβλία.
(misplaced possessive– intended meaning: My office is full of books)

54. Βαγγέλης ο γύρισε σπίτι κουρασμένος.
(misplaced determiner– intended meaning: Vangelis came back home tired)
